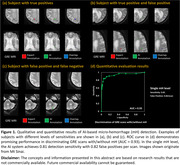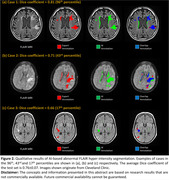# Utilizing Advanced Artificial Intelligence for Automated Detection and Segmentation of Amyloid‐Related Imaging Abnormality (ARIA)

**DOI:** 10.1002/alz.093974

**Published:** 2025-01-09

**Authors:** Long Xie, Paul A. Yushkevich, Sandhitsu R. Das, David A Wolk, Eli Gibson

**Affiliations:** ^1^ Siemens Healthineers, Princeton, NJ USA; ^2^ Perelman School of Medicine, University of Pennsylvania, Philadelphia, PA USA; ^3^ Penn Image Computing and Science Laboratory (PICSL), University of Pennsylvania, Philadelphia, PA USA; ^4^ Department of Neurology, University of Pennsylvania, Philadelphia, PA USA; ^5^ Siemens Heathineers, Princeton, NJ USA

## Abstract

**Background:**

The recent breakthrough in monoclonal antibody treatment for Alzheimer’s disease (AD) has ushered in a new phase in AD healthcare. However, associated amyloid‐related imaging abnormalities (ARIA) present a significant risk to patients, necessitating careful monitoring. Detection by radiologists can be challenging and may suffer from inconsistency. This study investigates the potential of advanced artificial intelligence (AI) for automated detection of micro‐hemorrhage (mH) in GRE MRI and ARIA‐like abnormal hyper‐intensity in FLAIR MRI, the two primary manifestations of ARIA.

**Methods:**

In a multi‐site clinical MRI dataset, we selected 399 GRE scans with micro‐hemorrhage as well as 161 FLAIR scans from stroke and posterior reversible encephalopathy syndrome (PRES) patients with ARIA‐like abnormal hyper‐intensity. Expert annotators, under the supervision of a radiologist, manually delineated micro‐hemorrhage and abnormal hyper‐intensity. An additional 2789 GRE scans of normal subjects were included to evaluate micro‐hemorrhage detection performance. 75% of the data was used to train two AI models to segment micro‐hemorrhage and abnormal hyper‐intensity respectively. Both AI models, adopting the U‐Net architecture, were fine‐tuned from a foundational model pretrained on another 5322 clinical MRI scans in a self‐supervised manner. Evaluation was conducted on the test set (the remaining 25%). The receiver operating characteristic (ROC) analysis was performed to investigate the discrimination of GRE scans with and without micro‐hemorrhage. Additionally, the sensitivity, and false positives of detecting individual micro‐hemorrhage were reported. For abnormal FLAIR hyperintensity, the Dice coefficient of the AI‐derived segmentation was computed.

**Results:**

The prototype discriminated GRE scans with and without micro‐hemorrhage with an ROC area under the curve of 0.93 (Figure 1‐d). At the single micro‐hemorrhage level, the AI system achieved a detection sensitivity of 0.81 with 0.82 false positives per scan. For abnormal FLAIR hyper‐intensity segmentation, our AI model achieved an average Dice coefficient of 0.76±0.07 (Figure 2). Qualitative results are presented in Figures 1 and 2.

**Conclusions:**

The findings demonstrate that the proposed AI systems, grounded in foundational AI techniques, can reliably detect micro‐hemorrhage from GRE scans and segment ARIA‐like abnormal FLAIR hyper‐intensity. These systems may play a crucial role in the automated detection and monitoring of ARIA, leading to enhanced patient care.